# Basic Test Framework for the Evaluation of Text Line Segmentation and Text Parameter Extraction

**DOI:** 10.3390/s100505263

**Published:** 2010-05-25

**Authors:** Darko Brodić, Dragan R. Milivojević, Zoran Milivojević

**Affiliations:** 1 Technical Faculty Bor, V.J. 12, University of Belgrade, 19210 Bor, Serbia; E-Mail: dbrodic@tf.bor.ac.rs; 2 Department of Informatics, Zeleni Bulevar 35, Mining and Metallurgy Institute, 19210 Bor, Serbia; E-Mail: dragan.milivojevic@irmbor.co.rs; 3 Technical College Niš, Aleksandra Medvedeva 20, 18000 Niš, Serbia; E-Mail: zoran.milivojevic@vtsnis.edu.rs

**Keywords:** OCR, document engineering, text line segmentation, text features, testing

## Abstract

Text line segmentation is an essential stage in off-line optical character recognition (OCR) systems. It is a key because inaccurately segmented text lines will lead to OCR failure. Text line segmentation of handwritten documents is a complex and diverse problem, complicated by the nature of handwriting. Hence, text line segmentation is a leading challenge in handwritten document image processing. Due to inconsistencies in measurement and evaluation of text segmentation algorithm quality, some basic set of measurement methods is required. Currently, there is no commonly accepted one and all algorithm evaluation is custom oriented. In this paper, a basic test framework for the evaluation of text feature extraction algorithms is proposed. This test framework consists of a few experiments primarily linked to text line segmentation, skew rate and reference text line evaluation. Although they are mutually independent, the results obtained are strongly cross linked. In the end, its suitability for different types of letters and languages as well as its adaptability are its main advantages. Thus, the paper presents an efficient evaluation method for text analysis algorithms.

## 1. Introduction

Printed text is defined by strong shape regularity. Its text lines have similar orientation and its skewness is also similar or equal, hence text orientation on same page is not variable. Descenders and ascenders from neighbor text lines are mostly disjoint and consequently, they do not interfere. Accordingly, text distances between lines are big enough to regularly split up text lines. Word in text lines are formed regularly, with similar distances and inter word spacing is decent.

Handwritten text is fully or partially cursive text. It tends to be multi-oriented and skewed. Text lines in handwritten documents are primarily curvlinear and close to each other. Descenders and ascenders from neighbor text lines are occasionally mixed up. Text distances between lines are close to each other, hence text lines run in to each other. Words in text lines are not formed regularly, so their distance is different. On the other hand, like printed text, handwritten text inter-word spacing is tolerable. Overall the appearance of skewed lines with different orientation and text lines close to each other make handwritten text less readable.

From the above, printed and handwritten text are characterized by their feature diversity. Hence, their text line segmentation as well as parameter extraction procedure can be quite dissimilar, although algorithms should fulfill these tasks for printed as well as for handwritten text.

Prior to text parameter extraction, text line segmentation should be done. It is an important step in document image processing. Although some text line detection techniques are successful in printed documents, processing of handwritten documents has remained a key problem in OCR [1,2]. Most text line segmentation methods are based on the assumptions that the distance between neighboring text lines is significant and that text lines are reasonably straight. However, these assumptions are not always valid for handwritten documents. Hence, text line segmentation is a leading challenge in document image analysis [3].

Upon completion of this process, the primary goal of OCR is the extraction of text parameters from optically scanned documents, so reference text line and skew rate identification is mandatory. Their validity is of major importance for any OCR process. There are various reasons for the appearance of multi-skewed lines in text, but two of them are the most common [1]: Firstly, some degree of misalignment of the document during the scanning process is unavoidable, but since all printed text lines in the scanned document are uniformly skewed, this way the reference text lines are almost parallel. Secondly, text lines in an original handwritten document are skewed differently due to specific individual handwriting habits, so handwriting text lines present different orientations, *i.e.*, they are multi-skewed. To enhance the ability of document analysis system, we need a robust algorithm for text line segmentation as well as for parameter extraction.

Many proposed algorithms have been evaluated by quite different test methods. In fact, these evaluation procedures are usually based on use of a custom text database as a test sample. Accordingly, testing result interpretation is quite dissimilar [4]. Hence, the establishment of the test framework for the evaluation of the document image processing algorithms is of great importance. This is precisely the task of this paper, and a basic method framework for the evaluation of the text line segmentation and text parameters extraction is proposed.

The paper is organized as follows: in Section 2 the test framework is presented. It is divided into two test groups. Each of them is completely described. Section 3 contains an examination and evaluation of the test framework procedure using an example of the specific algorithm. A Gaussian isotropic kernel is used as the basic test algorithm. Results are analyzed, examined and discussed. Conclusions are given in Section 4.

## 2. Evaluation Test Framework

The evaluation test framework for the text parameter extraction algorithm consists of a few text experiments. They are divided into two distinct groups:

1. Text line segmentation experiments,
2. Reference line and skew rate experiments.

Text line segmentation experiments are related to the algorithm’s ability to achieve segmentation of the text lines. Hence, these experiments are based on various multi-line sample texts. They incorporate the following tests:

- Multi-line text segmentation test,
- Multi-line waved text segmentation test,
- Multi-line fractured text segmentation test.

In contrast, the reference line and skew rate tests evaluate the algorithm’s competence for text line tracking. Therefore, they are based on single line sample texts as a reference. They include the following tests:

- Single line skew rate test,
- Single line waved text test,
- Single line fractured text test.

**Figure 1.**
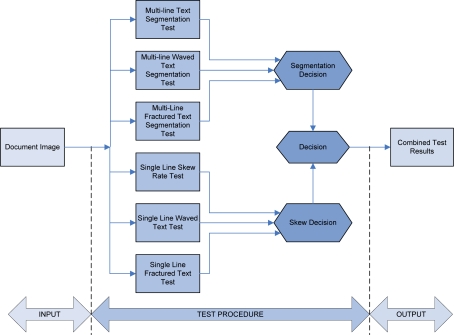
Integral framework test procedure.

A schematic diagram of the integral framework test procedure is shown in Figure 1. According to everything presented above, decision making is required at the end of the test procedure. Firstly, decision making is mandatory for the multi-line text segmentation experiments process. As a result of this decision, the sub-set values of the algorithm parameters are obtained. These parameter values are used as an optimization starting point. Further, results from single line text experiments are evaluated. These results narrow the algorithm optimization choice by creating its own parameters value sub-set. Although the test experiments are quite diverse, their results are inter-related. Hence, the last decision includes a new parameter sub-set values taking into account the all previously obtained parameter sub-set values. This final result represents the optimized parameter values.

### 2.1. Document text image

At the beginning of the test process, an original image is used. Assume that original image is continual function *f* (*x*, *y*). A document text image is obtained as a product of the original image scanning. Hence, the values of the coordinates (*x*, *y*) become discrete quantities. Now, the document text image is a digital text image represented by a matrix **D** with *M* rows, *N* columns, and intensity with *L* discrete levels of gray. *L* is the integer number from the set {0,…,255}. Hence, the intensity of matrix **D** is represented as [5]:

(1) D(r,c)=f(x,y)

where the origin of the function *f* (*x*, *y*) is point (*x*, *y*) = (0, 0), while the origin of the matrix **D** is (*r*, *c*) = (1, 1). Hence, row *r* ∈ {1,…,*M*} replaces *x* ∈ {0,…,*M*–1} and column *c* ∈ {1,…,*N*} replaces *y* ∈ {0,…,*N*–1}.

After applying intensity segmentation with binarization, the intensity function is converted into a binary intensity function given by:

(2) $$D_{\mathrm{bin}}(r,c)=\left\{ \begin{array}{c} 1\;\mathrm{for}\;D(r,c)\geq D_{\mathrm{th}}\\ 0\;\mathrm{for}\;D(r,c)<D_{\mathrm{th}} \end{array}\right.$$

where *D_th_* is given by the Otsu algorithm [6]. It represents a threshold sensitivity decision value.

**Figure 2.**
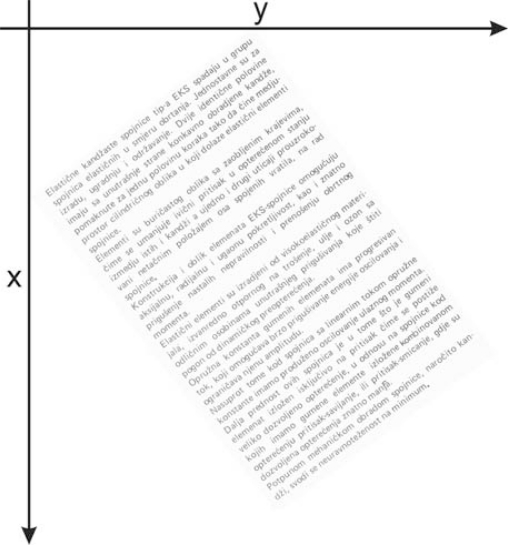
Document text image.

Now, extracted text lines are represented as a digitized document image by matrix **X** featuring *M* rows by *N* columns. Currently, the document text image is represented as a black and white image. It consists of the only black and white pixels. Each character or word consists of the only black pixels. Each pixel **X***_i,j_*, *i.e.*, **X**(*i, j*) is represented by the number of coordinate pairs such as:

(3) X(i,j)∈{0,255}

where *i* = 1,…,*M*, *j* = 1,…,*N* of matrix **X** [5]. In addition, value 0 represents black pixels, while value 1 from (2) converted in number 255 represents white pixels. This circumstance is shown by the document text image fragment in Figure 2.

### 2.2. Test procedure

#### 2.2.1. Multi-line text segmentation experiment

**Figure 3.**
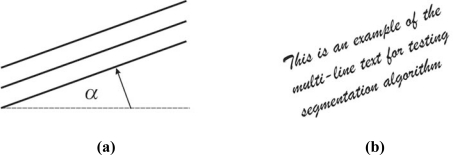
(a) Multi-line text referent line definition. (b) Multi-line text sample.

Algorithm quality examination consists of few text experiments representing the test procedure. In the first group of the experiments, text line segmentation quality is examined. These tests are significant because they are a prerequisite for obtaining the other text parameters. If segmentation experiment fails, then the examination of other features will be meaningless. Hence, its importance is critical. For this purpose, as the first experiment, a multi line text is used. Sample multi-line text with its skew angle parameter *α* is shown in Figure 3.

A number of existing text objects in a multi-line text image relate to the success of text segmentation. Hence, the less objects the better segmentation process, except the number may not be less than the number of text lines. As a quality measure, the root mean square error *RMSE_seg_* has been used. It is calculated as [7–9]:

(4) $${\mathrm{RMSE}}_{\mathrm{seg}}=\sqrt{\frac{1}{P}\sum_{k=1}^{P}{\left(O_{k,\mathrm{ref}}-O_{k,\mathrm{est}}\right)}^{2}}$$

where *k* = 1,…,*P* is the number of examined text samples, *O_k,ref_* is the number of referent objects in text, *i.e.*, number of text lines, and *O_k,est_* is the number of objects obtained in the text by the applied algorithm.

#### 2.2.2. Multi-line waved text segmentation experiment

**Figure 4.**
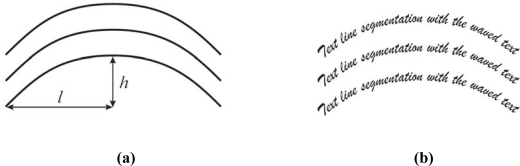
(a) Curved text referent line shape definition. (b) Curved multi-line text sample.

The second text line segmentation experiment is a multi-line curved text one. Sample text is formed as a group of text lines using a curved reference line for its basis. The reference line is defined by the parameter *ɛ* = *h/l*. Typically, *ɛ* is used from the set {1/8, 1/6, 1/4, 1/3, …}. A sample multi-line curved text for the experiment is shown in Figure 4.

Like in the previous segmentation test, the number of existing text objects after the algorithm is applied relates to the text segmentation quality. Again, as a quality measure, the root mean square error *RMSE_seg,wav_* has been used. It is calculated as [7–9]:

(5) $${\mathrm{RMSE}}_{\mathrm{seg},\mathrm{wav}}=\sqrt{\frac{1}{R}{\sum_{l=1}^{R}\left(O_{l,\mathrm{ref}}-O_{l,\mathrm{est}}\right)}^{2}}$$

where *l* = 1,…,*R* is the number of examined text samples, *O_l,ref_* is the number of referent objects in text, *i.e.*, number of text lines, and *O_l,est_* is the number of objects obtained in the text by the applied algorithm.

#### 2.2.3. Multi-line fractured text segmentation experiment

**Figure 5.**
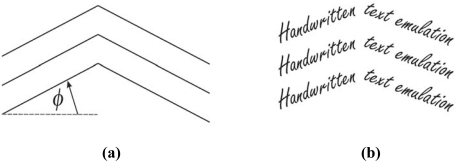
(a) Fractured text referent line slope definition. (b) Fractured multi-line text.

The last experiment in the first test group is a multi-line fractured text segmentation experiment. The sample text for this experiment is formed by using a reference fractured line as a basis. This fractured text reference line is defined by the slope angle *ϕ*, as a parameter. Typically, *ϕ* is used from the set {5°, 10°, 15°, 20°}. A sample multi-line fractured text for the last segmentation experiment is shown in Figure 5.

Again, the number of existing text objects relate to the text segmentation quality. The root mean square error *RMSE_seg,frac_* has been used. It is calculated as [7–9]:

(6) $${\mathrm{RMSE}}_{\mathrm{seg},\mathrm{frac}}=\sqrt{\frac{1}{Q}{\sum_{m=1}^{Q}\left(O_{m,\mathrm{ref}}-O_{m,\mathrm{est}}\right)}^{2}}$$

where *m* = 1,…,*Q* is the number of examined text samples, *O_m,ref_* is the number of referent objects in text, *i.e.*, number of text lines, and *O_m,est_* is the number of objects obtained in the text by the applied algorithm.

#### 2.2.4. Skew rate text experiment

**Figure 6.**
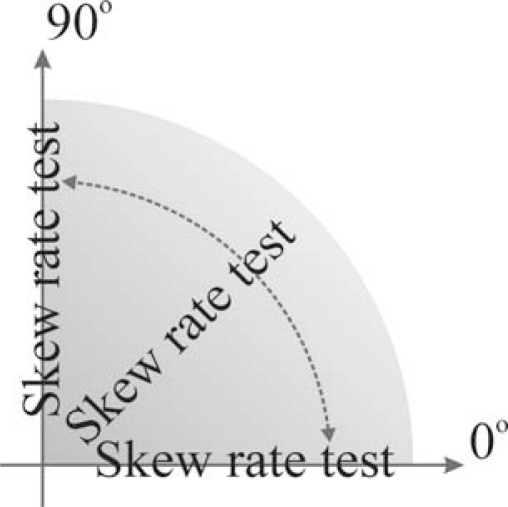
Sample text rotated up to 90° in 5° steps.

Further experiments belong in the second test group. The first of them, a skew rate test experiment, is mainly concerned with skew rate identification. It evaluates the algorithm’s performance in the skew tracking domain. Although, this experiment is primarily based on printed text, it is good prerequisite for testing handwritten text as well. In this test, a sample printed text rotated from 0° to 90° in 5° steps around the *x*-axis is used. This is presented in Figure 6.

The reference line of the test sample text is represented by:

(7) y=ax+b

After applying any algorithm to the sample text, reference text line estimation implies calculation of the average positions of only black pixels in every column of the document text image. It is calculated by [1,7,10]:

(8) $$x_{i}=\frac{\sum_{j=1}^{L}y_{j}}{L}\;\;\;\;\;\;\;\;\;\;i=1,\ldots ,K$$

where *x_i_* is the point position of calculated reference text line, *i* is the number of column position of the calculated reference text, *y_j_* is the position of black pixel in column *j* and *L* is the sum of black pixel number in a specified column *j* of an image.

After calculation, an image matrix with only one black pixel per column is obtained. It defines the calculated i.e. estimated reference text line as well as text line skewness. This reference text line forms a continuous or discontinuous line partly or completely “representing” the reference text line. To achieve a continuous linear reference text line, the least squares method is used. The function is approximated by a first-degree polynomial is given by:

(9) y=a′x+b′

Further, *ndp* represents the number of data points. It is used in the relation for calculating the slope *a’*, and the *y*-intercept *b’* as follows [8]:

(10) $$a^{\prime }=\frac{\sum y\sum \mathrm{xy}-\mathrm{ndp}\sum \mathrm{xy}}{{\left(\sum x\right)}^{2}-\mathrm{ndp}\sum x^{2}}$$

and:

(11) $$b\prime =\frac{\sum x\sum \mathrm{xy}-\sum y\sum x^{2}}{{\left(\sum x\right)}^{2}-\mathrm{ndp}\sum x^{2}}$$

For algorithm approximation and evaluation, a quantity called relative error [9] is important. The reference line hit rate *i.e.*, *RLHR* incorporates this quantity. It is defined as [7,10]:

(12) $$\mathrm{RLHR}=1-\frac{\Delta \beta }{\beta }=-\frac{\left|\beta_{\mathrm{est}}-\beta_{\mathrm{ref}}\right|}{\left|\beta_{\mathrm{ref}}\right|}$$

where *β_ref_* is the arc tangent from the origin (7) *i.e.*, *a* and *β_est_* is the arc tangent from estimate (9), *i.e.*, *a’*. Obviously, *RLHR* is equal to 1– the relative error [9]. Now, the root mean square error *RMSE_skew_* is calculated by [7–10]:

(13) $${\mathrm{RMSE}}_{\mathrm{skew}}=\sqrt{\frac{1}{S}{\sum_{n=1}^{S}\left(O_{n,\mathrm{ref}}-O_{n,\mathrm{est}}\right)}^{2}}$$

where *n* = 1,…,*S* is the number of examined text rotating angles up to 90°, *x_n,ref_* is *RLHR* for *β_est_* equal to *β_ref_*, due to normalization equal to 1, and *x_n,est_* is *RLHR*.

#### 2.2.5. Handwritten curved text experiment

**Figure 7.**
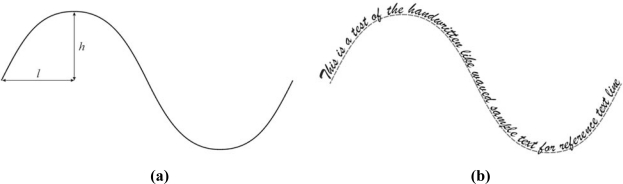
(a) Wavy text reference line slope and shape definition. (b) Wavy text sample.

The second experiment in this test group is primarily linked with handwritten text. Particularly, in this experiment a hypothetical reference text line is represented by a wavy line. This test examines the algorithm’s capability to follow a wavy reference text line. This wavy text sample as well as its definition is given in Figure 7.

Different types of wavy text can be examined. As can be seen, it is completely defined by the ratio *η* = *h/w* (see Figure 7a), where *h* represents height of the waved line, while *w* represents the half length of the wavy line. This experiment used the parameter set *η* = {1/8, 1/4, 1/2, 1}. Algorithm criteria quality and handwritten referent text line is measured and evaluated by the root mean square error *RMSE_wav_* calculated as [7–9]:

(14) $${\mathrm{RMSE}}_{\mathrm{wav}}=\sqrt{\frac{1}{T}{\sum_{o=1}^{T}\left(O_{o,\mathrm{est}}-O_{o,\mathrm{ref}}\right)}^{2}}$$

where *o* = 1,…,*T* is the number of examined text pixels, *i.e.*, columns of interest, *x_o,ref_* is pixel position of original referent text line in *o*-th column, and *x_o,est_* is pixel position of calculated, *i.e.*, estimated referent text line in *o*-th column.

#### 2.2.6. Handwritten fractured text experiment

**Figure 8.**
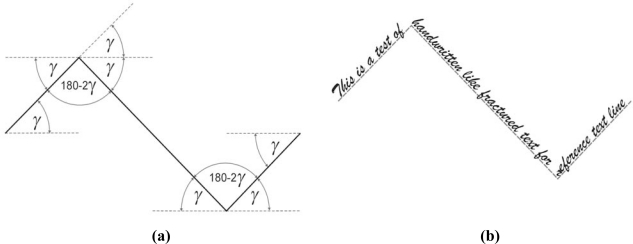
(a) Fractured text reference line slope definition. (b) Fractured text sample.

The next and the last experiment is also linked with handwritten text. A hypothetical reference text line is represented by a fractured line. This test examines the algorithm’s ability to follow a fractured text line which represents abrupt changes in direction. The fractured text sample is given in Figure 8. In the figure, *γ* is the slope angle of the first, second and third part of the fractured text line. It can be observed that the second and third part of text line is rotated by an angle of 2*γ* from previous part of reference text line at once. Hence, this test example is a rather extreme one.

This experiment is typically performed for *γ* from 5° to 25° in 5° steps around the horizontal *x*-axis. Again, the evaluated reference text line is evaluated by root mean square error (*RMSE*) method. Further, *RMSE_frac_* is calculated as [7–9]:

(15) $${\mathrm{RMSE}}_{\mathrm{frac}}=\sqrt{\frac{1}{U}{\sum_{p=1}^{U}\left(x_{p,\mathrm{est}}-x_{p,\mathrm{ref}}\right)}^{2}}$$

where *p* = 1,…,*U* is number of examined text pixels, *i.e.*, columns of interest, *x_p,ref_* is pixel position of original referent text line in *p*-th column, and *x_p,est_* is pixel position of calculated, *i.e.*, estimated referent text line in *p*-th column.

#### 2.2.7. Decision Making

Results obtained from different experiments during test procedure are inter-related. It should be noted that if the algorithm was examined and evaluated for text line segmentation as well as for text parameter extraction, then text line segmentation should be primary goal. Therefore, it is prerequisite for text parameters extraction such as reference text line and skew rate. This will be followed by the experiments for text parameter extraction and identification. Although, they are of second-rate importance compared to text line segmentation, their importance is evident at the next level of algorithm quality evaluation.

### 2.3. Combined test results

Combined test results represent merged results. Although, it is very similar to decision making, it is quite diverse from it. In fact, for the investigation of the algorithm under different parameters and restrictions, such merging of results leads to optimized parameter(s) value extraction. This way, an optimized subset of parameter(s) values is obtained. This process is invaluable for the algorithm evaluation as well as for obtaining any conclusions from it.

## 3. Test Example and Discussion

For the illustration, above test framework procedure will be examined using as an example the Gaussian isotropic kernel algorithm [7]. This algorithm will be just briefly explained. Its main task is expanding black pixel areas of text by scattering every black pixel in its neighborhood. This way, distinct areas that mutually separate text lines are established. Its primary purpose is joining only text elements from the same text line into the same distinct continuous areas. Gaussian probability function is taken as template that gives the probability of the random function. Consequently, it represents probability of the hypothetical expansion around every black pixel that represents a text element. Hence, around every black pixel, new pixels are non-uniformly dispersed. These new pixels have lower black intensity. Because the level of probability expansion relates to distance from black pixel, their intensity depends completely on their position *i.e.*, the distance from the original black pixel. Hence, these newly formed pixels are grayscale. Currently, document text image is represented by a grayscale image matrix. Thus, intensity pertains in level region {0,…,255}. Hence, after applying Gaussian isotropic kernel, equal to 2*K* + 1 in *x*-direction as well as in *y*-direction, text is scattered forming an enlarged area around it. Now, inside the kernel a “probability” sub area is formed using the radius 3*σ*, where *σ* represents standard deviation defining curve spread parameter. Converting all these pixels into black pixels as well as inverting image, forms the new black pixel expanded areas [7]. These areas are named boundary-growing areas.

**Figure 9.**
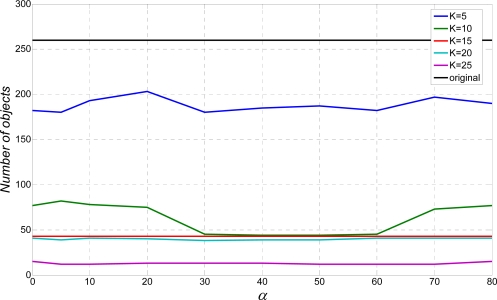
Number of the objects as a result of multi-line segmentation test.

**Figure 10.**
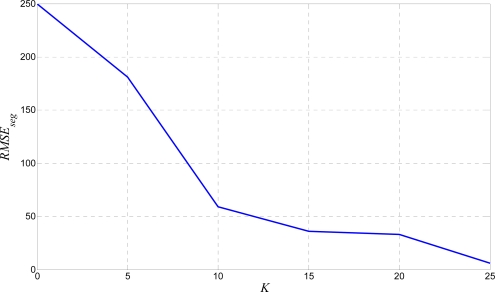
*RMSE_seg_* from the segmentation test results.

The main purpose of the testing is optimization of the algorithm parameters. In our example, parameter of interest is *K* that defines kernel size. Further, the algorithm will be examined and evaluated. Firstly, the algorithm is examined by a multi-line text segmentation test. The multi-line text sample is skewed by an angle *α* (see Figure 3). Gaussian kernel size is defined by *K* value (in pixels), which is used as parameter. Because of the size of the letters, *K* is used from the set {5, 10, 15, 20, 25}. Obtained results are presented in Figures 9 and 10.

Although it is not part of the “relevant” measurement test, but just for the illustration purposes, the number of segmentation objects for different *K* is shown in Figure 9. Parameter *K* = 0 represents situation without an applied algorithm. Obviously, a bigger *K* value leads to better segmentation results due to stretching of the original text. Still, too big a *K* will join different text lines.

**Figure 11.**
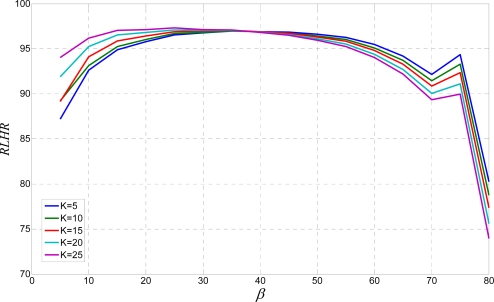
*RLHR* for text rotated by angle *β* from 0° up to 80°.

Similarly, the results presented in Figure 10 confirm that a big *K*, especially bigger than 15 leads to satisfactory segmentation results, while a small *K,* less than 10 are completely unacceptable. Hence, as a starting point, a *K* bigger than 15 is a good choice. Further, the algorithm is evaluated by a single line skew rate test. The obtained results are represented by the *RLHR* value from (12). They are shown in Figure 11.

**Figure 12.**
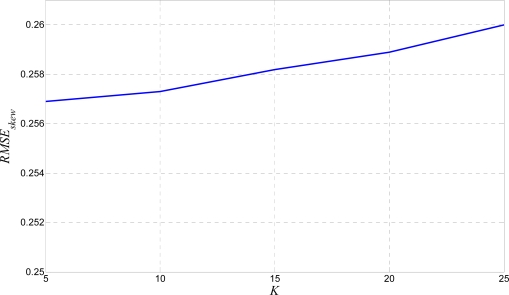
*RMSE_skew_* from the single line skew test.

This time, a bigger *K* is not an advantage. Hence, a medium size *K* like {15, 20} is the optimal value [7]. Scattering results from the previous test is represented by the *RMSE* method in Figure 12.

**Figure 13.**
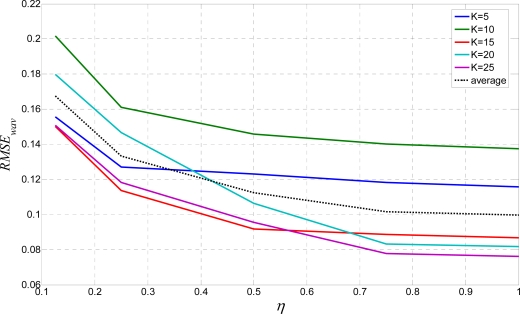
*RMSE_wav_* for the wavy text test.

Bigger *K* leads to slightly bigger *RMSE_skew_*. This is obvious because skewed reference line is more stretched by a bigger kernel which leads to slightly bigger scattering results. Finally, the algorithm is examined with a single line wavy text as well as a fractured text test. Algorithm criteria quality is measured by the *RMSE* method. Results for the wavy text test are shown in Figure 13.

**Figure 14.**
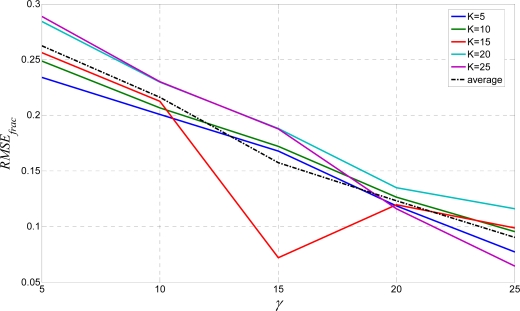
*RMSE_frac_* for the fractured text test.

From Figure 7a, the ratio *η* = *h/w* is the severe element. Response to the sample wavy text is optimal for a *K* value chosen from the set {15, 20, 25} that confirms their candidacy for the optimal values. Finally, results for the fractured text test are shown in Figure 14. The most promising *RMSE_frac_* value for this test is for *K* = 15. Hence, from all the above results, after intersection decision making, the optimal values for *K* are 15 or 20.

From the obtained and presented measurement results, it is obvious that the results of above tests are quite sufficient for the evaluation of the algorithm quality in the domain of the text segmentation and feature extraction. Hence, they can represent a basic test framework for the evaluation of the text line segmentation and text parameters extraction.

## 4. Conclusions

The paper describes the proposal of a basic test framework for the evaluation of text feature extraction algorithms. All previous algorithm evaluation procedures were custom oriented. However, the proposed test framework is the first step toward testing generalization in the domain of document image processing algorithms. It consists of two groups of experiments. In the first and the most important group, text line segmentation experiments are included. These tests measure text line segmentation algorithm quality. They incorporate three various multi-line text experiments. Single line skew rate test belongs in the second group of the test framework. Its task is algorithm performance evaluation of the skew rate tracking success. Further tests in this group of experiments are primarily linked to “handwritten text”. They consist of single line wavy and fractured text tests. These tests examine an algorithm’s ability to follow wavy and fractured text reference lines. Results obtained from all test experiments are inter-related. Hence, after decision making and results merging, optimized values of the algorithm parameters are extracted. This way, an optimized subset of parameters values is obtained. Hence, this process is invaluable for algorithm evaluation as well as for making any conclusions about it. In the end, its suitability for different types of letters and languages as well as its adaptability is a strong advantage.

## Appendix

**Appendix 1.** Multi-line text segmentation test results.

**Number of text objects**						
***α***	***K* = 5**	***K* = 10**	***K* = 15**	***K* = 20**	***K* = 25**	***K* = 0**
**0°**	182	77	43	41	15	260
**5°**	180	82	43	39	12	260
**10°**	193	78	43	41	12	260
**20°**	203	75	43	40	13	260
**30°**	180	45	43	38	13	260
**40°**	185	44	43	39	13	260
**50°**	187	44	43	39	12	260
**60°**	182	45	43	41	12	260
**70°**	197	73	43	41	12	260
**80°**	190	77	43	41	15	260

***RMSE_seg_***	**181.05**	**59.22**	**36.00**	**33.02**	**6.01**	-

**Appendix 2.** Single line skew rate test results.

**Reference line hit rate (*RLHR*)**					
***β***	***K* = 5**	***K* = 10**	***K* = 15**	***K* = 20**	***K* = 25**
**5°**	87.19%	89.24%	89.13%	91.88%	94.05%
**10°**	92.63%	93.14%	94.10%	95.24%	96.20%
**15°**	94.89%	95.26%	95.89%	96.53%	97.05%
**20°**	95.80%	96.02%	96.43%	96.81%	97.11%
**25°**	96.53%	96.68%	96.89%	97.10%	97.32%
**30°**	96.76%	96.81%	96.95%	97.06%	97.14%
**35°**	96.96%	96.97%	97.03%	97.04%	97.07%
**40°**	96.91%	96.90%	96.91%	96.84%	96.83%
**45°**	96.84%	96.75%	96.72%	96.59%	96.48%
**50°**	96.62%	96.44%	96.32%	96.11%	95.94%
**55°**	96.27%	96.02%	95.83%	95.53%	95.25%
**60°**	95.48%	95.09%	94.82%	94.40%	94.02%
**65°**	94.19%	93.72%	93.28%	92.70%	92.19%
**70°**	92.15%	91.47%	90.88%	90.06%	89.34%
**75°**	94.34%	93.30%	92.36%	91.12%	89.97%
**80°**	80.24%	78.73%	77.35%	75.57%	73.95%

***RMSE_skew_***	**0.2569**	**0.2573**	**0.2582**	**0.2589**	**0.2600**

**Appendix 3.** Single line waved text test results.

***RMSE_wav_***					
***η = h/w***	***K* = 5**	***K* = 10**	***K* = 15**	***K* = 20**	***K* = 25**
**1/8**	0.1556	0.1271	0.123	0.1183	0.1158
**1/4**	0.2017	0.1611	0.1459	0.1402	0.1376
**1/2**	0.1502	0.1138	0.0919	0.0887	0.0869
**3/4**	0.1798	0.1467	0.1064	0.0832	0.0819
**1**	0.1509	0.1184	0.0955	0.0779	0.0761

***RMSE_avg_***	**0.1676**	**0.1334**	**0.1125**	**0.1017**	**0.0997**

**Appendix 4.** Single line fractured text test results.

***RMSE_frac_***					
***γ***	***K* = 5**	***K* = 10**	***K* = 15**	***K* = 20**	***K* = 25**
**5°**	0.234	0.2009	0.1677	0.1185	0.0772
**10°**	0.2487	0.2067	0.1721	0.1264	0.0956
**15°**	0.2563	0.2124	0.0719	0.1197	0.0986
**20°**	0.2842	0.2296	0.1882	0.1349	0.1160
**25°**	0.2888	0.2302	0.1876	0.116	0.0645

***RMSEavg***	**0.2624**	**0.2160**	**0.1575**	**0.1231**	**0.0904**

## References

[1] Basu S., Chaudhuri C., Kundu M., Nasipuri M., Basu D.K. (2006). Text Line Extraction from Multi-Skewed Handwritten Documents. Pattern Recognition.

[2] Amin A., Wu S. Robust Skew Detection in mixed Text/Graphics Documents.

[3] Likforman Sulem L., Zahour A., Taconet B. (2007). Text Line Segmentation of Historical Documents: A Survey. IJDAR.

[4] Razak Z., Zulkiflee K., Idris M.Y.I., Tamil E.M., Noor M.N.M., Salleh R., Yaakob M., Yusof Z.M., Yaacob M. (2008). Off-Line Handwriting Text Line Segmentation: A Review. IJCSNS.

[5] Gonzalez R.C., Woods R.E. (2002). Digital Image Procesing.

[6] Otsu N. (1979). A Threshold Selection Method from Gray-level Histograms. IEEE Trans. Syst., Man, Cybern.

[7] Brodić D., Milivojević Z. Using Anisotropic Gaussian Window for Printed and Handwritten Text Parameters Extraction.

[8] Bolstad W.M. (2005). Introduction to Bayesian Statistics.

[9] Terell G.R. (1999). Mathematical Statistics: A Unified Introduction.

[10] Brodić D., Milivojević Z., Camarinha-Matos L.M., Pereira P., Ribeiro L. (2010). An Approach to Modification of Water Flow Algorithm for Segmentation and Text Parameters Extraction. Emerging Trends in Technological Innovation.

